# Triplex ELISA for Assessing Durability of *Taenia solium* Seropositivity after Neurocysticercosis Cure

**DOI:** 10.3201/eid2907.230364

**Published:** 2023-07

**Authors:** Nina L. Tang, Theodore E. Nash, Madelynn Corda, Thomas B. Nutman, Elise M. O’Connell

**Affiliations:** National Institutes of Health, Bethesda, Maryland, USA

**Keywords:** neurocysticercosis, cysticercosis, parasites, zoonoses, ELISA, antibody, serologic response, EITB, seropositivity, Taenia solium, United States

## Abstract

Neurocysticercosis prevalence estimates often are based on serosurveys. However, assessments of *Taenia solium* seropositivity durability in patients with various neurocysticercosis types are lacking. We optimized a triplex serologic ELISA by using synthetic GP50, T24H, and Ts18var3 antigens for *T. solium*. We used that assay to test sequential serologic responses over several years after neurocysticercosis cure in 46 patients, 9 each with parenchymal or ventricular neurocysticercosis and 28 with subarachnoid disease. Triplex results were concordant with 98% of positive and 100% of negative enzyme-linked immunoelectrotransfer blots. Eight years after neurocysticercosis cure, 11.1% of patients with parenchymal, 47.3% with subarachnoid, and 41.7% with ventricular disease were still seropositive. Median time to seroreversion after cure in this cohort in a *T. solium* nonendemic area was 2 years for parenchymal disease, 4 years for ventricular disease, and 8 years for subarachnoid disease. Our findings can inform epidemiologic models that rely on serosurveys to estimate disease burden.

Neurocysticercosis is a parasitic disease caused by infection with larval cysts of the pork tapeworm, *Taenia solium*. Serosurveys are crucial tools for understanding the epidemiology of *T. solium* infection and populations at risk for neurocysticercosis. However, even in highly disease-endemic communities, concurrent neuroimaging reveals that few *T. solium*–seropositive neurocysticercosis patients have viable cysts at any given time (*1*,*2*). The discrepancy between seropositivity and evidence of central nervous system infection is likely the result of a combination of factors. One contributor is certainly persisting antibody response after intracranial cysts have resolved. However, the kinetics of serologic reactivity of patients after neurocysticercosis cure has not been demonstrated in follow-up beyond 1 year after treatment (*3*). Because serologic responses to any antigen can be boosted by repeat exposure, assessing long-term responses in a population that has left an endemic area and has little possibility of reexposure can be particularly useful.

The reference standard serologic diagnostic assay for neurocysticercosis is the enzyme-linked immunoelectrotransfer blot (EITB). EITB uses *T. solium* glycoproteins purified with lentil lectin to detect *Taenia*-specific antibodies, and positivity to any of 7 glycoproteins of interest is considered a positive result. EITB has a universally high sensitivity for detecting viable neurocysticercosis cysts, except in the case of a single parenchymal cyst. However, EITB testing is extremely labor intensive, requires parasite material, depends on expertise to accurately interpret the results, and has low throughput (*4*). Previous studies identified the 7 glycoproteins detected by EITB, which encompass 3 families of proteins, GP50, T24, and 8kD (*5*–*8*). Using QuickELISA (Immunetics Inc., https://immunetics.com) and a synthetic or recombinant antigen from each family has been previously reported to have high sensitivity for each component (*9*). That finding contrasts with ELISAs that use crude *Taenia* antigens and have reduced sensitivity and specificity (*10*,*11*).

We assessed the sensitivity of a slightly different combination of antigens representing the 3 families of proteins (GP50, T24H, and Ts18var3) used in the EITB assay in a traditional ELISA by using samples known to be EITB-positive or EITB-negative. We subsequently assessed the durability of seroreactivity after cure in a well-characterized group of patients with parenchymal, ventricular, and subarachnoid neurocysticercosis followed longitudinally in a nonendemic setting.

## Methods

### Samples

To define assay cutoffs, we obtained samples from 2 sources. First, we obtained samples from patients with known neurocysticercosis enrolled in an Institutional Review Board–approved neurocysticercosis natural history protocol (no. NCT00001205) at the National Institutes of Health Clinical Center, (Bethesda, Maryland, USA). All persons in that protocol provided written consent and were confirmed to be serologically positive by EITB performed at the Centers for Disease Control and Prevention (Atlanta, Georgia, USA). A second source of serum and plasma was deidentified samples left over from clinical testing that would otherwise have been discarded and that had quantitative PCR–detectable levels of circulating *T. solium* antigen (*12*), *T. solium* DNA, or both (*13*). Patients with positive results by those assays had a large burden of viable *T. solium* and were universally positive by EITB (*14*), here referred to as putative positives. To establish the optimal cutoffs for our triplex assay, we used serum or plasma from 100 known EITB-positive or putative positive patients and 52 persons from an endemic area, 29 of whom were known to be EITB-negative and 23 of whom were healthy blood bank controls without epidemiologic exposures. We collected samples from EITB-positive patients <18 months after their positive EITB. The pool of neurocysticercosis patients included cases of various cyst locations and represented a diverse range of geographic exposure (Table).

**Table:** Demographics of a cohort of neurocysticercosis patients who underwent serial ELISA testing for reactivity to *Taenia solium* antigens GP50, T24H, and Ts18var3 to assess seropositivity after cure*

Characteristic	Value
Sex	
M	28
F	18
Age at cure, y	
19–29	15
30–60	30
>60	1
Region of birth	
Asia	4
Central America	29
Mexico	8
South America	4
United States	1
Neurocysticercosis type†	
Parenchymal	
Single cyst	3
>1 cyst	6
Extraparenchymal	
Subarachnoid	28
Ventricular	9
Total cohort size	46
Median length of follow-up, y (range)	
Parenchymal	8.5 (4–17.5)
Subarachnoid	6.6 (1.2–16.6)
Ventricular	8.4 (2.6–12.1)


*Values are no. patients except as indicated. †Patients were classified by their most severe form of disease: subarachnoid, ventricular, then parenchymal.

Patients from the neurocysticercosis natural history protocol that were serologically positive during active infection by the triplex assay and had banked samples with at least 2 years of follow-up after cure also underwent sequential sample testing. Cure for subarachnoid neurocysticercosis was defined as the first timepoint at which *T. solium* DNA was undetectable in cerebrospinal fluid (CSF) (n = 11) by qPCR (*13*) or *T. solium* antigen testing (*12*). For patients who did not have CSF available for testing (n = 17), we defined cure as the date anthelmintics were stopped; all patients in that group had >3 years of clinical follow-up with brain magnetic resonance imaging (MRI) after anthelmintics were discontinued. For patients with ventricular disease, we defined time of cure as the date on which the lesion was calcified on computed tomography (CT) imaging or susceptibility-weighted MRI images or when T2-weighted MRI sequences became dark, or at the time of surgical resection if the cyst was removed. Patients with parenchymal disease had to have noncalcified cysts at study entry to be included in this cohort. We defined time of cure for parenchymal disease as the date that imaging first demonstrated calcification or complete lesion resolution. Most patients were able to provide samples from both before and after cure. Year after cure was calculated as the date of serum collection minus the date of cure and rounded to the closest year.

### Triplex *T. solium* Assay

The triplex *T. solium* assay used recombinant T24H and GP50 proteins. T24H (8) was expressed in a High Five (BTI-Tn-5B1-4) (Kempbio, http://www.kempproteins.com) baculovirus expression system (*8*). GP50 was produced (Genscript, https://www.genscript.com) as previously described (*7*). The synthetically produced 74 amino acid polypeptide Ts18var3 (LifeTein, https://www.lifetein.com) was derived from a *T. solium* DNA sequence (Genbank accession no. AF098075) (*5*,*6*) (Appendix). We tested additional proteins, TsRS1 (*6*) and NC3 (*15*), but preliminary tests on those 2 proteins were not as sensitive as the 3 proteins used (data not shown), and we did not pursue those 2 proteins further.

### Statistical Analysis

We used Prism 9 (Graphpad, https://www.graphpad.com) to perform statistical analyses. We produced receiver operating characteristic (ROC) curves by using the Wilson-Brown method for sensitivity and specificity and set 95% CIs. We calculated the J-index at various signal-to-noise cutoffs by adding sensitivity and specificity and subtracting 1. To compare the seroreactivity of different disease types to T24H, GP50, and Ts18var3, we used Kruskal-Wallis analysis of variance and Dunn multiple comparisons tests with adjusted p values. We used Fisher exact tests and Baptista-Pike odds ratios (ORs) to determine the specificity of various seroreactivity patterns for different forms of neurocysticercosis. We used Fisher exact tests to determine differences in seropositivity at cure among neurocysticercosis groups. We used nonparametric Wilcoxon matched pairs signed-rank tests to compare the seroreactivity to T24H, GP50, and Ts18var3 at the time of cure and 4 years after cure. We used Kaplan-Meier simple survival analyses to describe the annual prevalence of seroreversion after cure and log-rank Mantel-Cox tests to analyze differences between survival curves.

## Results

### Sensitivity and Specificity of *T. solium* Triplex Assay 

**Figure 1:**
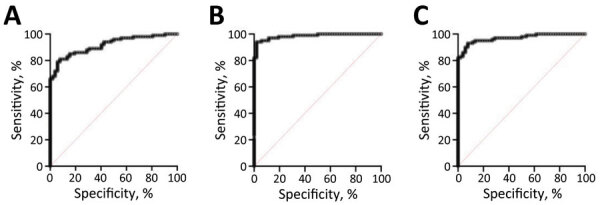
Sensitivity and specificity of a triplex assay to determine durability of *Taenia solium* seropositivity after neurocysticercosis cure. The assay combines 3 families of *T. solium* antigens: A) T24H; B) GP50; and C) Ts18var3. Receiver operating characteristic curves were produced by testing serum from 52 persons who were *T. solium–*negative and 100 neurocysticercosis patients with positive enzyme-linked immunoelectrotransfer blot results: 17 with parenchymal disease, 16 with ventricular disease, 35 with subarachnoid disease, and 32 putative positives (patients with detectable levels of circulating *T. solium* antigen, *T. solium* DNA, or both).

**Figure 2:**
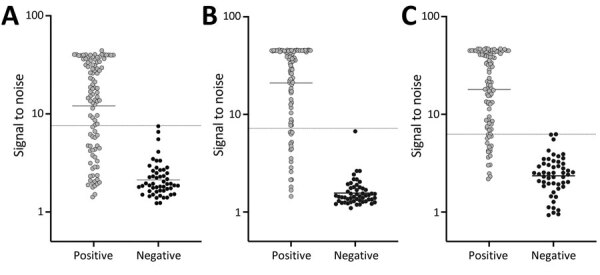
Signal-to-noise results of a triplex assay to determine durability of *Taenia solium* seropositivity after neurocysticercosis cure. The assay combines 3 families of *T. solium* antigens: A) T24H; B) GP50; and C) Ts18var3. Samples from neurocysticercosis positive and negative persons were compared; each circle represents 1 person. Horizontal bars indicate geometric mean; dotted horizontal lines indicate signal-to-noise cutoffs that provided the highest sensitivity while maintaining 100% specificity for each protein.

For all proteins, we compared the sensitivity and specificity produced by various signal-to-noise cutoffs determined using ROC curves (Figure 1). We defined sensitivity as the percent positive by the triplex assay out of the 100 samples known to be positive by EITB or antigen testing. We defined specificity as the percent of the 52 samples known to be EITB-negative that were also negative by the triplex assay. The J-index cutoffs for signal-to-noise were 3.547 (J = 0.7331) for T24H, 2.742 (J = 0.9208) for GP50, and 4.019 (J = 0.8531) for Ts18var3. Those cutoffs resulted in sensitivity of 81% (95% CI 72%–88%) and specificity of 92% (95% CI 82%–97%) for T24H, sensitivity of 94% (95% CI 88%–97%) and specificity of 98% (95% CI 90%–100%) for GP50, and sensitivity of 93% (95% CI 86%–97%) and specificity of 92% for Ts18var3 (95% CI 82%–97%). In addition to the J-index, we used ROC curves for each protein to determine cutoffs that yielded 100% specificity. This approach resulted in signal-to-noise thresholds of 7.6 for T24H, 7.206 for GP50, and 6.261 for Ts18var3. With those cutoffs, each protein independently achieved moderate sensitivity, 66% for T24H and 82% for both GP50 and Ts18var3 (Figure 2).

**Figure 3:**
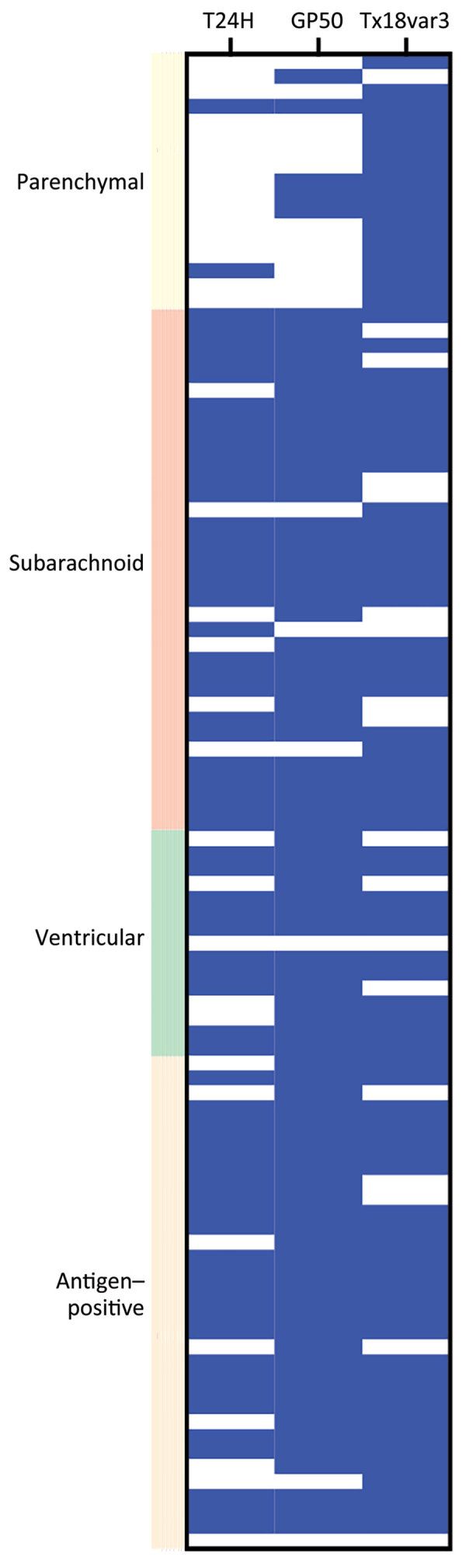
Comparison of positive and negative results of a triplex assay to determine durability of *Taenia solium* seropositivity after neurocysticercosis cure. The assay combines 3 families of *T. solium* antigens: T24H, GP50, and Ts18var3. The graph shows results for each antigen from 100 persons with and without neurocysticercosis, by disease type. Blue indicates positive results; white indicates negative results.

To improve the diagnostic capacity of the individual proteins, we combined the results of each assay and defined *T. solium* positivity as positivity to any of the 3 proteins. Using this approach, the sensitivity of the combined triplex assay exceeded that of any individual protein (Figure 3). When we used cutoffs that yielded 100% specificity, the triplex assay achieved a sensitivity of 98% (95% CI 93%–100%), a major improvement over the sensitivities of each assay in isolation, demonstrating the power of this triplex approach.

### Differential Seroreactivity to Antigens 

**Figure 4:**
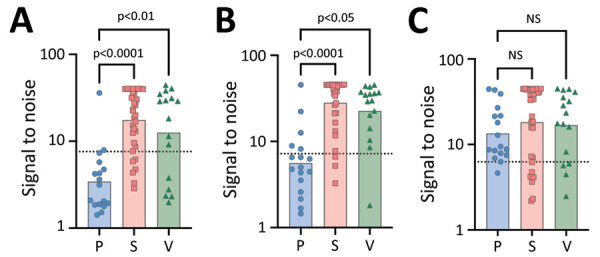
Positivity patterns per cyst location for a triplex assay to determine durability of *Taenia solium* seropositivity after neurocysticercosis cure. The assay combines 3 families of *T. solium* antigens: A) T24H; B) GP50; and C) Ts18var3. Graphs display signal-to-noise reactivity to T24H, GP50, and Ts18var3 by neurocysticercosis disease type. We assessed differential reactivity to *T. solium* antigens in samples from 68 neurocysticercosis-positive persons, 17 with parenchymal, 16 with ventricular, and 35 with subarachnoid neurocysticercosis. Dotted horizontal lines indicate 100% specificity cutoff each protein. NS, not statistically significant; P, parenchymal; S, subarachnoid; V, ventricular.

In addition to binary detection of *Taenia*-specific antibodies, we found the triplex assay provided information on disease type by assessing the differential reactivity of serum to the assays’ components. We noted no difference in reactivity (H2) between patients with subarachnoid and ventricular disease for T24H (H2 adjusted p>0.9999), GP50 (H2 adjusted p = 0.3676), or Ts18var3 (H2 adjusted p>0.9999), suggesting that extraparenchymal disease drives reactivity to all 3 proteins (Figure 4). We also saw no significant difference in seroreactivity toward Ts18var3 between patients with parenchymal and subarachnoid disease (H2 adjusted p = 0.5396) or parenchymal and ventricular disease (H2 p>0.9999).

**Figure 5:**
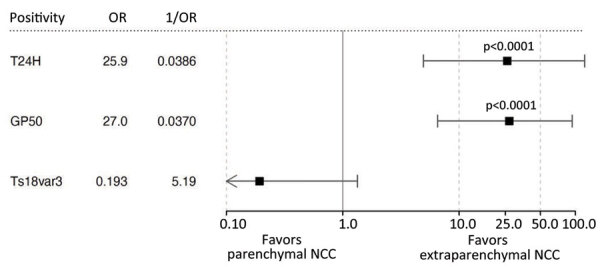
Odds ratios of seropositivity of a triplex assay to determine durability of *Taenia solium* seropositivity after neurocysticercosis cure. The assay combines 3 families of *T. solium* antigens: T24H, GP50, and Ts18var3. We assessed seropositivity in extraparenchymal and parenchymal disease. Error bars indicate 95% CIs; arrows indicate that the end of a CI exceeds the range of the graph axis. NCC, neurocysticercosis; 1/OR, reciprocal odds ratio; OR, odds ratio.

However, the divergent patterns of seroreactivity between extraparenchymal and parenchymal cases became clear when comparing reactivity to T24H and GP50. Subarachnoid patients exhibited higher reactivity than parenchymal patients to both T24H (H2 adjusted p<0.0001) and GP50 (H2 adjusted p<0.0001). Similarly, ventricular patients were also significantly more reactive than parenchymal patients to T24H (H2 adjusted p = 0.0046) and GP50 (H2 adjusted p = 0.0108). Odds ratio analysis using Fisher exact tests and Baptista-Pike ORs demonstrated the predictive capacity of those seroreactivity patterns; seropositivity to T24H resulted in 25.91 (95% CI 4.931−120.1; p<0.0001) times the odds of extraparenchymal over parenchymal disease and positivity to GP50 resulted in 27.00 (95% CI 6.531–93.71; p<0.0001) times the odds in the same direction (Figure 5). Meanwhile, positivity to only Ts18var3 resulted in nonsignificant odds of 5.189 (95% CI 0.7467–58.76; p = 0.1574) in favor of parenchymal over extraparenchymal neurocysticercosis.

**Figure 6:**
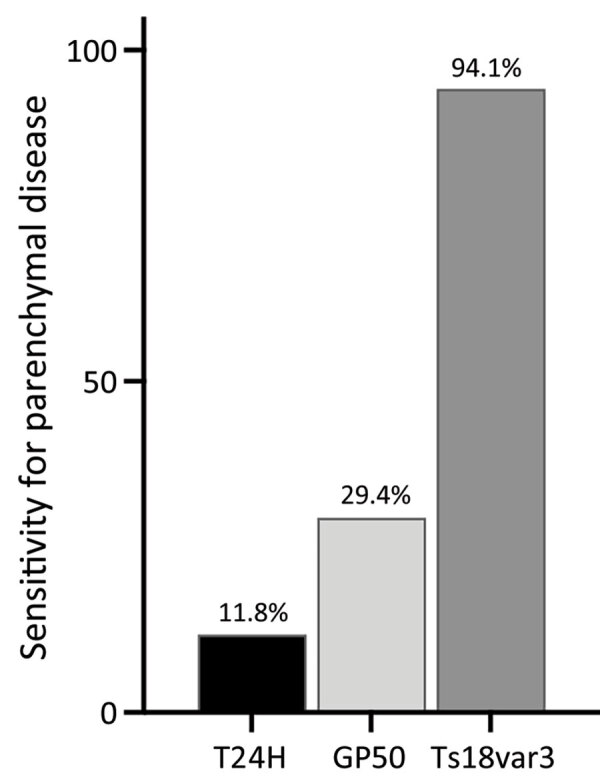
Sensitivity for parenchymal disease of a triplex assay to determine durability of *Taenia solium* seropositivity after neurocysticercosis cure. The assay combines 3 families of *T. solium* antigens: T24H, GP50, and Ts18var3. The graph shows that sensitivity is primarily dependent on Ts18var3; numbers at tops of bars indicate percentages of parenchymal cases (n = 17) found to be positive by each protein.

Although positivity to Ts18var3 did not confer any statistically significant predictive capacity to the triplex assay, it was the main driver of the triplex assay’s sensitivity for parenchymal neurocysticercosis. When we used only Ts18var3 with the 100% specificity cutoff of 6.261 signal-to-noise, we detected 94.1% (16/17) of patients with parenchymal disease, compared with 11.8% (2/17) when we only used T24H and 29.4% (5/17) when we only used GP50 (Figure 6). Therefore, to maximize detection of parenchymal disease, Ts18var3 is a necessary component of the assay.

### Persistence of Seropositivity after Neurocysticercosis Cure

To evaluate the persistence of seropositivity after cure, we longitudinally collected serum from 46 neurocysticercosis patients and evaluated samples by using the triplex *T. solium* assay. The patient cohort included 9 persons with active parenchymal neurocysticercosis, all of whom subsequently had calcified disease; 28 subarachnoid patients; and 9 ventricular patients. The median follow-up time was 8.5 (range 4–17.5) years for parenchymal disease, 6.6 (range 1.2–16.6) years for subarachnoid disease, and 8.4 (range 2.6–12.1) years for ventricular disease (Table).

**Figure 7:**
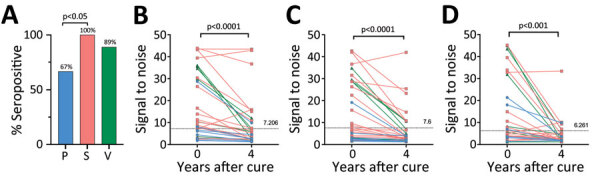
*Taenia solium* seropositivity over time by a triplex assay to determine durability of *T. solium* seropositivity after neurocysticercosis cure. The triplex assay combines 3 families of *T. solium* antigens: T24H, GP50, and Ts18var3. A) Seropositivity at time of cure. B–D) Seropositivity at year 4 by neurocysticercosis disease type: B) parenchymal (n = 9); C) subarachnoid (n = 28); and D) ventricular disease (n = 9). Line colors in panels B–D correspond to bar colors in panel A. Horizonal dotted lines indicate the 100% specificity cutoff used for that protein. Statistically significant differences in seropositivity were seen between patients with parenchymal disease (67%) and subarachnoid disease (100%) (p = 0.011), but not for ventricular disease (89%), at the time of cure. Patients seropositive at time of cure (parenchymal n = 6, subarachnoid n = 28, ventricular n = 8) underwent testing of paired samples at time of cure (day 0) and 4 years after cure. For each subgroup, reactivity to GP50, T24H, and Ts18var3 showed statistically significant decreases by year 4. P, parenchymal; S, subarachnoid; V, ventricular.

**Figure 8:**
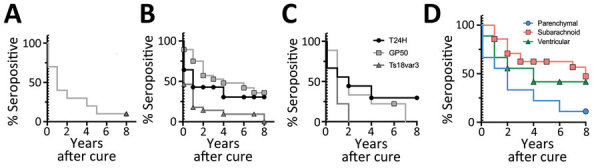
Degradation of *Taenia solium* seropositivity over time by a triplex assay to determine durability of *T. solium* seropositivity after neurocysticercosis cure. The assay combines 3 families of *T. solium* antigens: T24H, GP50, and Ts18var3. Kaplan-Meier survival curves show seropositivity by disease type for each protein over 8 years after neurocysticercosis cure. A) Parenchymal disease; B) subarachnoid disease; C) ventricular disease; D) all disease types. Time 0 represents time during treatment when all subjects were seropositive; dotted vertical line indicates time of cure. Symbols indicate years containing censored data for each disease type. The sample size for patients with parenchymal disease testing positive to T24H, GP50, or both, at cure were too few to plot. Therefore, these curves were excluded in this analysis. Log-rank Mantel-Cox analysis found significant differences in seroreversion in Ts18var3 compared with T24H and GP50 for both subarachnoid (p = 0.03) and ventricular disease (p = 0.04). Log-rank Mantel-Cox analysis for all survivors (D) demonstrates significant differences between the curves (p = 0.025); 11.1% of patients with parenchymal disease, 47.3% with subarachnoid disease, and 41.7% with ventricular disease were seropositive 8 years after cure.

At the time of cure, 67% of patients with parenchymal, 100% with subarachnoid, and 89% with ventricular disease were seropositive (Figure 7, panel A). In the first 4 years after cure, we noted all groups had statistically significant reductions in seroreactivity toward T24H (p<0.0001), GP50 (p<0.0001), and Ts18var3 (p = 0.0002) (Figure 7, panels B–D). This finding suggests a reduction in *T. solium* antibodies after disease resolution. However, the rate at which reactivity decreases to the individual antigens of the triplex assay is different. Kaplan-Meier survival analysis showed that, by 4 years after cure, 80% of patients with parenchymal, 90.5% with subarachnoid, and 100% with ventricular disease were no longer reactive to Ts18var3 (Figure 8, panels A–C). Insufficient numbers of patients with parenchymal disease were available to perform survival analysis for T24H and GP50. However, 4 years after cure, 69.4% of patients with subarachnoid disease seroreverted to T24H and 52% seroreverted to GP50. Meanwhile, 70% of patients with ventricular disease seroreverted to T24H and 78% seroreverted to GP50 over the same period. Log-rank Mantel-Cox analysis showed a significant difference in annual seroreversion between T24H, GP50, and Ts18var3 in both subarachnoid (p = 0.03) and ventricular patients (p = 0.04), and reactivity to Ts18var3 was the first to decline, then GP50 and T24H.

We used additional Kaplan-Meier survival analysis to determine the probability of complete seroreversion for each disease type, meaning that patients would achieve seronegativity to all proteins. That analysis demonstrated significant differences in seroreversion over time (p = 0.025) among the 3 different categories of neurocysticercosis. Median seropositivity was 2 years after cure for parenchymal disease, 8 years for subarachnoid disease, and 4 years for ventricular disease (Figure 8, panel D). Overall seropositivity at year 8 after cure was 11% for parenchymal disease, 47.3% for subarachnoid disease, and 41.7% for ventricular disease.

## Discussion

Although brain imaging is required to diagnose neurocysticercosis, serologic testing often confirms the diagnosis, and *T. solium* serosurveys often are performed to assess the burden of *T. solium* exposure in a population. We optimized a serologic ELISA by using 3 proteins in a triplex assay. The triplex *T. solium* assay was able to detect 98% of EITB-positive or antigen-positive cases and agreed 100% with EITB test negativity. Although recombinant T24H has been reported to have a high sensitivity and specificity when used in an ELISA for serologic detection (*9*), our study did not find T24H was an essential driver of overall positivity. In fact, of the 98 samples that were positive by the optimized triplex assay, 97 would still have been positive by relying only on GP50 and Ts18var3 without sacrificing specificity. This finding suggests that future diagnostic assays might be able to rely on only 2 antigens, but because neurocysticercosis involving a single parenchymal lesion has diminished seroreactivity, a larger scale study in a population more representative of those patients will be needed to verify that finding (*16*). In addition, EITB banding patterns have been reported to be somewhat different in small assessments in Asia (*17*). In ROC curve development for this study, we had 5 patients from countries in Asia, namely India, South Korea, and Laos. All those patients were *T.* solium–positive by the triplex assay without exception to any antigen. High quality evidence from serologic assessments in Asia also showed that reactivity to T24 and to the 8kDa family, of which Ts18var3 is a member, was maintained (*17*,*18*). However, because of the small representation of patients from Asia, formal validation of our triplex assay should be undertaken in endemic areas of Asia before deploying it in that setting.

In this group of patients, responsiveness to GP50 and T24H greatly increased the odds that the patient had extraparenchymal disease. In a previous study that analyzed EITB assay banding patterns, positivity to GP50 or T24 was associated with intraparenchymal infections (*19*). In that same study, reactivity to GP50, T24, and the 8kDa protein family were observed more frequently in extraparenchymal disease and patients with multiple intraparenchymal lesions (*19*). However, those differences likely are a result of the different platforms used for antibody detection. Such differences include the sensitivity in detecting antibodies to various antigens because of the different methods and because the positivity threshold for ELISA is artificially set by choosing a signal-to-noise ratio that maximizes sensitivity and specificity. EITB also might be limited by what can be visually observed in grading reactivity.

Clarifying the kinetics of seroreactivity to *T. solium* infection in neurocysticercosis patients is vital for determining the clinical utility these tests and epidemiologic studies that involve their use (*3*,*20*). Serial serosurveys without concurrent brain imaging in Peru and Ecuador have shown that, over the course of a year, ≈30% of serologically positive patients will revert to negative (*21*,*22*). Another study in Peru followed patients with parenchymal neurocysticercosis after therapy and demonstrated that only 7% of all treated patients became seronegative 1 year after treatment, and only 23% (3/13) of cured patients became seronegative (*3*). Here, we studied patients that had >1 positive serologic response during active disease and assessed what happened over time. By the time of cure, determined by CSF antigen negativity, calcification, or surgical removal, depending on disease type and sample availability, only 33% (3/9) of patients with parenchymal disease, 11% (1/9) with ventricular disease, and none (0/28) with subarachnoid disease seroreverted. The time parameters of our study are not completely comparable to the studies from Peru and Ecuador because it can take >1 year from the time of treatment for an intraparenchymal cyst to start to calcify, which we used as a criterium for cure in this study. However, we used cure to standardize the durability of seropositivity because in some cases viable cysts begin to die before treatment is begun, and some patients require multiple treatment courses. Regardless of the timeframe used for assessing seroreversion, serologic negativity is unreliable for determining disease activity in neurocysticercosis, as others have also demonstrated (*3*), and should not be used to assess treatment efficacy nor disease activity.

Our data show a major decrease in antibody reactivity to each antigen in the triplex assay for all neurocysticercosis disease states over time. However, the overall durability of seropositivity was striking; the median seropositivity for patients with parenchymal disease was 2 years, for subarachnoid disease was 8 years, and for ventricular disease was 4 years. Those differences appear to be driven by the difference in the kinetics of disappearance of the antibodies to the individual antigens. Reactivity to Ts18var3 was the most short-lived for each disease type. However, Ts18var3 is the main driver of positivity among patients with parenchymal disease; thus, the overall seropositivity for those with parenchymal disease was shorter. By comparison, patients with ventricular disease were reactive to Ts18var3 for a median of 1 year after cure, but >50% of patients with subarachnoid disease had already seroconverted to Ts18var3 at 1 year after cure.

A few aspects of this study should be considered when interpreting these data in a larger epidemiologic context. Our population is skewed toward more heavily infected patients, and most of the included patients had extraparenchymal neurocysticercosis. Our parenchymal disease cohort was relatively small, and most patients had >1 cyst. However, those data are particularly useful for demonstrating durability of seropositivity among patients with extraparenchymal disease, and nearly half of those patients were still seropositive 8 years after cure. That finding is notable because extraparenchymal disease is likely underdiagnosed, and the true prevalence likely is unknown and higher than previously appreciated (*23*). Moreover, symptoms of extraparenchymal disease are often not captured when screening populations by history because patients without parenchymal cysts do not have seizures, and a history of headaches is very nonspecific. Finally, CT imaging often misses extraparenchymal cysts and cannot be relied on for making that diagnosis. Thus, the contribution of extraparenchymal disease to the seropositivity of a population remains an area of active investigation. We did not address the contribution of cysts outside the central nervous system to overall seropositivity or the duration of seropositivity in those patients.

In conclusion, seroreversion in neurocysticercosis patients is often delayed for years after cure. We described the kinetics of serologic responses against 3 diagnostic antigens for *T. solium* from neurocysticercosis diagnosis to cure in persons who likely did not have further exposure to the parasite. These data could help inform epidemiologic models that rely on serosurveys to estimate disease burden. 

Appendix Additional information on a triplex ELISA for assessing durability of *Taenia solium* seropositivity after neurocysticercosis cure.
