# Supplementary material for: Triplex ELISA for Assessing Durability of Taenia solium Seropositivity after Neurocysticercosis Cure
Source: Emerg Infect Dis. 2023 Jul;29(7):1340–8. doi: 10.3201/eid2907.230364 (PMC10310364; doi:10.3201/eid2907.230364)
Supplement: Appendix — Additional information on a triplex ELISA for assessing durability of Taenia solium seropositivity after neurocysticercosis cure. [file 23-0364-Techapp-s1.pdf]

Article DOI: https://doi.org/10.3201/eid2907.230364

EID cannot ensure accessibility for supplementary materials supplied by authors. Readers who have difficulty accessing supplementary content should contact the authors for assistance.

Triplex ELISA for Assessing Durability of *Taenia solium* Seropositivity after Neurocysticercosis Cure

Appendix

Supplemental Data

Ts18var3 Peptide Sequence

FVVAVSAEETKPKCDANSTKKEIEYIHNWFFHDDPIGKQIAQLAKNWNETVQEAKGEIR
ASLAEYCRGLKNKTA, C-terminal Amidation

T24H Expressed in Baculovirus Expression System (KempBio)

Recombinant protein sequence:

YRHDFVRLVGKEMQEAIQELQSKRLSGSDPTLKALEELQAKLKCCGGVGPSDWRVAPP
SCCGKESGSCTSPYQTGCAEAMYNEMKDSALAFG

GP50 (17–276) Expressed in Baculovirus Expression System (GenScript)

Recombinant protein sequence:

MLLVNQSHQGFNKEHTSKMVSAIVLYVLLAAAAHSAFAVVSTSSENAPKMWGSRVIGK
PSGPSDTMSYEYNDNYRTVLINDSVLGTMSIKRNQCMLWETKPWGEPCNIFPGYVNITL
NNVTAQKIMEMDEITARPRVASTTFFVPHCNFTKPAPGEVDVWTSFPLSRFVKDTPWFR
VDFAVGGANYDSTATFDINATSLCFWRGTKLLHKGAEFCTDMVKDESADLRVFRGVFP
RKTNISRESFAFAGLKTALTVSIDYSQSGISPEVADCKQYAKVKDLSTLVATMPAYATKT
STGNHHHHHH

Supplemental Methods

T24, GP50, and Ts18var3 were diluted with phosphate-buffered saline (PBS) to coat flat-bottom Immulon 4 HBX treated 96-well plates overnight at 4°C; T24H and Ts18var3 were used at 1 μg/mL and GP50 at 0.1 μg/ml. Plates were washed using the BioTek 405 Touch Microplate Washer (BioTek; Winooski, VT) for 6 cycles with 300 μL of wash buffer (PBS + 0.03% Tween 20) and blocked for 45 minutes at 37°C in blocking buffer (PBS + 0.05% Tween 20 + 5% bovine serum albumin (BSA)). Again, plates were washed for 6 cycles with 300 μL of wash buffer. Serum was diluted in ELISA diluent (PBS + 0.05% Tween 20 + 1% BSA) 1:50 for T24H, 1:100 for GP50, and 1:10 for Ts18var3 and incubated for 45 minutes at 37°C. Again, plates were washed for 6 cycles with 300 μL of wash buffer. Alkaline phosphatase conjugated AffiniPure goat anti-human IgG Fcγ fragment specific antibody (Jackson ImmunoResearch; West Grove, PA) was diluted 1:1,000 in ELISA diluent and incubated for 45 minutes at 37°C. Again, plates were washed for 6 cycles with 300 μL of wash buffer. Secondary antibody staining was visualized by incubating with 1 mg/mL alkaline phosphatase substrate in sodium carbonate buffer at room temperature and protected from light for 30 minutes. Optical density was determined at 405 nanometers by Molecular Devices SpectraMax i3 using the SoftMax Pro 6.5.1 software (Molecular Devices; San Jose, CA). Seroreactivity was evaluated by calculating the signal-to-noise of optical density readouts. All samples were tested in duplicate with positive and negative controls and blanks on each plate. Seropositivity was defined based on cutoffs for each protein that preserved 100% specificity for *T. solium*.
